# Whole genome sequencing analysis of *Plasmodium vivax* using whole genome capture

**DOI:** 10.1186/1471-2164-13-262

**Published:** 2012-06-21

**Authors:** A Taylor Bright, Ryan Tewhey, Shira Abeles, Raul Chuquiyauri, Alejandro Llanos-Cuentas, Marcelo U Ferreira, Nicholas J Schork, Joseph M Vinetz, Elizabeth A Winzeler

**Affiliations:** 1 Biomedical Sciences Program, University of California, San Diego, La Jolla, CA, USA; 2 Biomedical Sciences ProgramDepartment of Genetics, University of CaliforniaThe Scripps Research Institute, San Diego, La Jolla, CA, USA; 3 The Scripps Translational Science Institute, The Scripps Research Institute, La Jolla, CA, USA; 4 Division of Biological Sciences, University of California, San Diego, La Jolla, CA, USA; 5 Department of Medicine, Division of Infectious Disease, University of California, San Diego, La Jolla, CA, USA; 6 Alexander von Humboldt Institute of Tropical Medicine, Universidad Peruana Cayetano Heredia, Lima, Peru; 7 Department of Parasitology, Institute of Biomedical Sciences, University of São Paulo, São Paulo, Brazil; 8 Department of Molecular and Experimental Medicine, The Scripps Research Institute, La Jolla, CA, USA; 9 Genomics Institute of the Novartis Research Foundation, San Diego, CA, USA

**Keywords:** Malaria

## Abstract

**Background:**

Malaria caused by *Plasmodium vivax* is an experimentally neglected severe disease with a substantial burden on human health. Because of technical limitations, little is known about the biology of this important human pathogen. Whole genome analysis methods on patient-derived material are thus likely to have a substantial impact on our understanding of *P. vivax* pathogenesis and epidemiology. For example, it will allow study of the evolution and population biology of the parasite, allow parasite transmission patterns to be characterized, and may facilitate the identification of new drug resistance genes. Because parasitemias are typically low and the parasite cannot be readily cultured, on-site leukocyte depletion of blood samples is typically needed to remove human DNA that may be 1000X more abundant than parasite DNA. These features have precluded the analysis of archived blood samples and require the presence of laboratories in close proximity to the collection of field samples for optimal pre-cryopreservation sample preparation.

**Results:**

Here we show that in-solution hybridization capture can be used to extract *P. vivax* DNA from human contaminating DNA in the laboratory without the need for on-site leukocyte filtration. Using a whole genome capture method, we were able to enrich *P. vivax* DNA from bulk genomic DNA from less than 0.5% to a median of 55% (range 20%-80%). This level of enrichment allows for efficient analysis of the samples by whole genome sequencing and does not introduce any gross biases into the data. With this method, we obtained greater than 5X coverage across 93% of the *P. vivax* genome for four *P. vivax* strains from Iquitos, Peru, which is similar to our results using leukocyte filtration (greater than 5X coverage across 96% ).

**Conclusion:**

The whole genome capture technique will enable more efficient whole genome analysis of *P. vivax* from a larger geographic region and from valuable archived sample collections.

## Background

The global burden of *Plasmodium vivax* is being increasingly reevaluated as more fatal cases are identified and drug resistant strains are discovered [1,2]. Despite the fact that 2.85 billion people live in *P. vivax* endemic areas, there is a substantial lack of knowledge surrounding the mechanisms of biological features unique to *P. vivax,* constraining the ability to design appropriate control strategies.

The fact that *P. vivax* exclusively invades reticulocytes impairs the development of a reliable, long-term *in vitro* culture method, a technique that has been available for the study of *P. falciparum* for over 30 years [3]. While some progress has been made in establishing *P. vivax* culture in the laboratory, the lack of a reproducible *in vitro* culture method prevents basic laboratory manipulations, such as genetic crosses, and has limited the types of questions that can be answered about *P. vivax* biology.

The advent of low-cost whole genome technologies allows direct analysis of *P. vivax* field populations, without the need for *in vitro* culture. With the completion of the *P. vivax* reference genome as well as the publication of the first *P. vivax* resequencing project [4], single nucleotide variants (SNV) are now being identified that can be used to track parasite populations and investigate parasite population structure on both the regional and global levels. In addition, new whole genome sequencing technologies allow for sequencing hundreds of samples from different geographic locations and thus take advantage of the thousands of natural genetic crosses that occur over time and in the context of parasite movements among regions under different epidemiological contexts (reverse genetics). Using signatures in the genome left by these natural crosses, investigators will be able to identify regions of the genome under selection and, potentially, the genes involved in *P. vivax* virulence, drug resistance, and immune evasion.

A critical barrier to the whole genome analysis of *P. vivax* is the ability to obtain sufficient quantities of high quality parasite genomic DNA free of human nucleic acid contamination. Current protocols for obtaining parasite DNA for whole genome studies from *P. falciparum* field isolates consist of culture adapting the isolated parasites and passaging them for 3–4 weeks. This intermediate step achieves two things: one, it expands the parasite population allowing for isolation of a larger quantity of DNA and, two, it removes human leukocytes containing contaminating DNA. Since there is no reliable culture method to propagate *P. vivax in vitro*, alternative methods have to be designed to work with nucleic acids from *P. vivax* field samples.

To address the two issues of low quantities of parasite DNA and human DNA contamination, the standard method adopted by the *P. vivax* research community is leukocyte filtration using ion-exchange columns followed by whole genome amplification (WGA) [4,5]. This current method of obtaining *P. vivax* DNA from field samples is only feasible when the patient blood samples are collected in close proximity to a field laboratory because of the need to filter out the leukocytes before they lyse. This logistical issue precludes the collection of field samples from remote areas where *P. vivax* is endemic and thus limits our understanding of the population genetics of *P. vivax*. In addition, there are many samples that were collected before leukocyte depletion became a standard technique. As of now these samples cannot be analyzed *via* whole genome sequencing prohibiting the use of these samples to study ofhow *P. vivax* has evolved over time.

Here we demonstrate the feasibility of analyzing *P. vivax* field samples without on-site leukocyte filtration using an in-solution hybridization capture method [6,7]. By modifying the whole genome capture protocol designed for *P. falciparum* by Melnikov *et al.*[8], we show that Sal1 reference genomic DNA can be used to create whole genome baits, which can then be used to extract *P. vivax* genomic DNA from the contaminating human DNA in both frozen samples and mock blood spots. After the whole genome extraction of *P. vivax* DNA and subsequent whole genome sequencing, greater than 90% of the *P. vivax* assembled genome (~22 million bases) can be confidently assigned a genotype, or “called.” Our whole genome sequencing results are equivalent to previous results published using the leukocyte filtration protocol, and we, therefore, propose that because of its much easier application in the field, whole genome capture is a superior method of analyzing large numbers of *P. vivax* field samples from diverse geographic areas.

## Results

### Synthesis of whole genome baits

**Figure 1:**
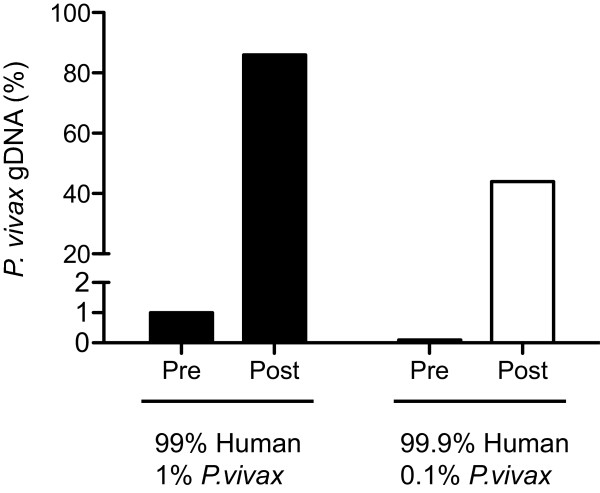
**Enrichment of Sal1 DNA using whole genome baits.** Mock *P*. *vivax* infections were created by combining Sal1 reference DNA and human DNA. These samples were then whole genome captured using the Sal1 derived baits. Pre and Post bars refer to the percent *P. vivax* DNA in the mock sample before and after whole genome capture.

We created whole genome baits (WGB) using *in vitro* transcription with Sal1 genomic DNA as the template. Briefly, Sal1 genomic DNA was fragmented to an average of 200 bp and a T7 promoter sequence was ligated onto the fragment ends. *In vitro* transcription was then conducted in the presence of biotinylated dUTP, creating biotinylated RNA baits. The WGB were initially tested on two mock Sal1 infections: one created by combining 1% Sal1 DNA with 99% human DNA and a second created by combining 0.1% Sal1 DNA and 99.9% human DNA. The baits were able to enrich these mock samples from 1% *P. vivax* DNA to 86% *P. vivax* DNA and from 0.1% *P. vivax* DNA to 44% *P. vivax* DNA (Figure 1). Additional WGB can be created through subsequent *in vitro* transcription reactions.

**Whole genome capture and sequencing of*P. vivax*field samples**

We performed a whole genome capture protocol (see Methods) on five field samples (SA-94, SA-95, SA-96, SA-97, SA-98) collected in Iquitos, Peru during the 2010 transmission season using the WGB derived from Sal1 genomic DNA. Patient blood samples were centrifuged after collection with the serum and Buffy coat subsequently removed. Erythrocytes were then frozen at −80 °C and shipped back to the United States for further analysis. For three samples (SA-96, SA-97, SA-98), DNA was isolated from the frozen red blood cell pellet. The parasitemias for these three samples were within the expected range for *P. vivax* infection, which is substantially lower than *P. falciparum* infection (Table 1). In order to assess the proportion of *P. vivax* DNA in our starting material we analyzed the bulk genomic DNA using qPCR. The percent of *P. vivax* DNA present after enrichment with whole genome capture was determined by whole genome sequencing (see below) (Table 1). For the remaining two samples (SA-94 and SA-95), 125 ul of erythrocytes were placed on Whatman FTA nucleic acid filter paper, stored for 2 weeks at room temperature to create mock blood spots, and then DNA was isolated using a standard extraction protocol.

**Table 1:** ***P. vivax*genomic DNA yield from whole blood samples using whole-genome capture**

Isolate	Parasitemia			% *P. vivax* gDNA		
	**Parasites/μl blood**	**Parasites/RBC (%)**	**Bulk DNA yield (μg)**	**Pre-WGC**	**Post-WGC**	**Fold enrichment**
SA-96	2,300	0.04%	3.2	0.53%	52.89%	99.8
SA-97	2800	0.05%	7	1.34%	60.52%	45.2
SA-98	8700	0.16%	7.6	3.95%	78.40%	19.8
SA-94	6100	0.11%	2.4	na	80.13%	na
SA-95	1800	0.03%	1.7	na	20.25%	na
IQ07^a^	na	na	na	41.87%	na	na
Acre3^b^	na	na	na	1.42%	na	na

Abbreviations: RBCs, red blood cells; WGC, whole-genome capture; na, not available.

^a^positive control - leukocyte filtered, ^b^negative control.

Genomic DNA recovered after whole genome capture was sequenced using the Illumina HiSeq 2000 platform and 4.7 - 6.3 billion bases of data were obtained per sample. The sequencing reads were then aligned to the Sal1 reference using BWA [9], and sequencing and alignment characteristics were generated using Picard and the Genome Analysis Toolkit (GATK) [10]. We compared the sequencing statistics from the whole genome captured samples to the genome sequence of IQ07. This strain from Iquitos, Peru was collected in the 2007 transmission season and had previously been prepared by leukocyte filtration and sequenced by our lab [4]. Since IQ07 is the only published sequencing data for a *P. vivax* sample taken directly from a patient, we considered the sequencing analysis of the IQ07 strain as our positive control. We also obtained sequencing data for Acre3, a *P. vivax* strain collected in Acre, Brazil, which had been neither leukocyte filtered nor enriched using whole genome capture. Acre3 served as our negative control.

After aligning the sequencing reads, PCR duplicates, which arise during the final PCR amplification step of both library preparation methods, were marked to reduce false coverage. We identified PCR duplicates by finding those sequencing pairs that aligned to the exact same location in the genome and had the same insert size. The percentage of PCR duplicates ranged from 12.1% to 46.2% and was higher than the percentage of PCR duplicates identified in IQ07 (1.86%) or Acre3 (4.38%) (Table 2). The percentage of PCR duplicates was in line with the percentage of PCR duplicates seen in exome capture studies [11]. After identifying PCR duplicates, sequencing reads from each sample were locally realigned around indels and areas of high entropy, and all base quality scores were then recalibrated based on empiric sample-reference mismatch data.

**Table 2:** Sequencing statistics for whole-genome capture samples

	Number of bases sequenced (billion)		% PCR duplicates		% genome covered by 5 or more reads
Isolate		% *P. vivax* gDNA		Coverage (X)	
IQ07^a^	2.3	41.87%	1.86%	35.16	95.33%
Acre3^b^	1.4	1.42%	4.38%	0.77	0.80%
SA-94^c^	6.0	80.13%	13.60%	150.31	97.21%
SA-95^c^	5.3	20.25%	46.17%	21.89	84.73%
SA-96^c^	4.7	52.89%	32.29%	63.11	93.35%
SA-97^c^	5.2	60.52%	26.37%	86.75	94.37%
SA-98^c^	6.3	78.40%	12.14%	160.44	96.51%

^a^positive control - leukocyte filtered, ^b^negative control, ^c^whole-genome capture.

Following clean up of the aligned reads, we compared the percent of reads that aligned to the *P. vivax* reference from the whole genome capture samples to the controls. Of the 57.9 million sequencing reads obtained for IQ07, 42% of the reads aligned to the Sal1 reference (Table 2). For Acre3, the negative control, only 1.42% of the 23.5 million sequencing reads aligned to the Sal1 reference. In contrast to the *P. vivax* field samples analyzed here, >90% of reads obtained from sequencing laboratory reared *P. falciparum* (with no human contaminating DNA) align to the *P. falciparum* reference.

For the whole genome captured samples the percentage of reads that mapped to the Sal1 reference ranged from 20% to 80% with 4 out of 5 samples analyzed having a higher percentage of *P. vivax* DNA than in IQ07 and all samples showing a much higher percentage of *P. vivax* DNA than Acre3 (Table 2). In addition, the percentage of reads that aligned to the Sal1 reference was directly related to the parasites per ul present in the original patient sample and thus the variance in the whole genome capture result for both frozen samples and mock blood spots is a function of the starting parasite gDNA load (Table 1). Future studies using the whole genome capture protocol would benefit from prioritizing samples for sequencing based upon the patients parasitemia at time of sample collection to limit non-specific binding during the capture protocol.

### Whole genome and chromosome coverage analysis of whole genome captured samples

After identifying all the reads that mapped to the *P. vivax* reference genome, we filtered out low quality reads (mapping quality (MQ) < 29) and low quality bases (base quality (BaseQ) < 20) from those reads that aligned. Both MQ and BaseQ are Phred scaled scores indicating, respectively, the accuracy of the alignment as given by BWA and the accuracy of the base call as determined by the empiric sample-reference mismatch rate taking into account known variants. These filtration steps are necessary to ensure that machine errors and alignment errors do not bias the conclusions drawn from the data and the resulting set of high quality bases was used for all subsequent downstream analysis discussed below.

**Figure 2:**
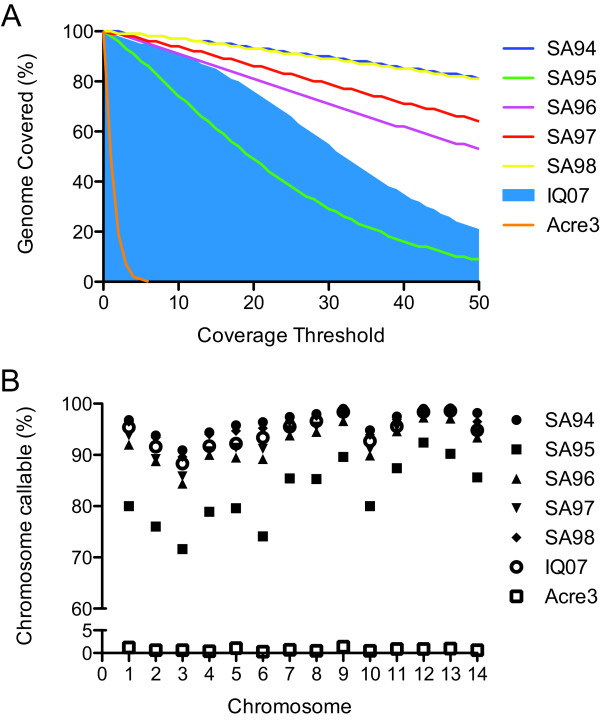
***P. vivax*genomic coverage after whole genome capture.** (**A**) Percent of the *P. vivax* genome covered by various coverage thresholds for the five whole genome capture samples, the IQ07 positive control (shaded region), and the Acre3 negative control (orange). (**B**) The percent of each chromosome that was able to be confidently genotyped, ie callable, for the whole genome captured samples compared to the IQ07 positive control (upper scale). The Acre3 negative control is presented on the lower scale.

First, genome wide sequencing coverage was computed from the set of high quality aligned bases for all whole genome captured samples and was compared with the IQ07 positive control and the Acre3 negative control. IQ07 was sequenced to a genome wide depth of 34.16X. Genome wide coverage by high quality bases for the captured samples ranged from 21.89X to 160.44X and was positively correlated to the percent of the reads that mapped to the *P. vivax* reference (Table 2). In addition, the percentage of the genome covered by different sequencing depths (5X, 10X, *etc.*) was directly correlated to the genome wide coverage achieved and is presented for all whole genome captured samples along with the positive (shaded area) and negative (orange line) controls (Figure 2A).

Next we evaluated the number of bases that were “callable,” *i.e.* able to have a confident genotype assigned to them. We defined “callable” for this haploid organism as being covered by five or more bases all of which have a BaseQ of 20 or above (99% accurate). Using this cutoff we predict one incorrectly called genotype in 10 billion genotype calls or one incorrect genotype call in approximately 454 malaria genomes analyzed by sequencing. In the IQ07 strain 95.3% of the entire genome was callable and in the Acre3 negative control only 0.80% of bases were callable. Of the five genome captured samples, greater than 90% of all bases were able to be called in four of the samples with the worst performing fifth sample, SA-95, still having 84.73% of the genome callable.

While genome wide the percentage of callable bases was high, we next looked at the percentage of callable bases on the individual chromosome level in order to identify gross biases in the capture technique (Figure 2B). Again we used sequencing data from IQ07 as the standard. All samples prepared by whole genome capture exhibited the same trend of percentage of callable bases per chromosome as IQ07, but a slight overall bias to certain areas of the genome was identified requiring more sequencing to analyze these regions.

### %GC content accounts for the sequencing bias between the two techniques

We next examined those regions of the genome that performed poorly in the whole genome capture technique as compared to the leukocyte filtration protocol. The bias identified is correlated directly with %GC. The mean %GC of *P. vivax* is 45% and both the leukocyte filtration method and the whole genome capture protocol are able to efficiently sequence regions of mean %GC content (Additional file 1: Figure S1 and Figure S2 (A)). In addition, both sequence preparation methods are poor at sequencing areas of substantially lower %GC (< 30%).

Where the two methods diverge is sequencing regions of higher %GC (>50%). The capture method is only approximately half as efficient at sequencing these regions as the leukocyte filtration method (Additional file 1: Figure S1 and Figure S2 (B)). This effect is seen across all 14 chromosomes and the differential bias is seen most prominently around the centromere on account of these regions containing a higher %GC on average than the whole genome.

Aside from %GC, the bias seen in the capture samples does not correlate with any other genomic feature including SNVs, chromosomal location (except the centromere because of the higher %GC), particular gene families, or gene rich/poor regions.

### Quantity of sequencing data required for *P. vivax* resequencing projects

We next investigated the amount of sequencing data from either the traditional library preparation or the whole genome capture protocol needed to call a certain percentage of the genome. Using the data from SA-94, for which we had initially obtained 150X genome wide coverage, we randomly downsampled the original data set to obtain downsampled data sets from 5X to 50X genome wide coverage at 5X intervals. Only properly mapped read pairs were included in this analysis and the reads from a pair were either both chosen or both excluded. The same downsampling strategy was used on IQ07 to obtain downsampled data sets from 5X to 30X coverage at 5X intervals. We also analyzed the full 34X IQ07 dataset.

**Figure 3:**
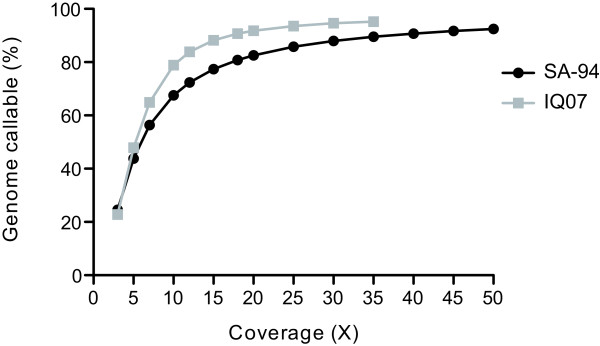
**Confidently genotyped loci at various sequencing depths.** SA-94 (black) and IQ07 (gray) were randomly downsampled and the mean percent of the genome that is callable of 5 randomly produced data sets for each coverage threshold is presented.

At 20X genome wide coverage, 82.52% of the SA-94 genome is covered by five or more reads and therefore callable (Figure 3). At the same genome wide coverage, 91.8% of the IQ07 genome is callable. This difference in callable genome percentage at the same level of genome wide coverage indicates that certain areas of the genome are more efficiently captured than others leading to uneven coverage across the genome. As explained above the regions that are underrepresented in sequencing libraries prepared using whole genome capture are those areas with high %GC. This effect is slight but reproducible with all of our whole genome captured samples. As the genome wide coverage increases the discrepancy in callable bases between the two library preparation methods decreases. At 30X genome wide coverage, 87.96% of the SA-94 genome is callable compared to 94.6% of the IQ07 genome.

While regions of higher %GC are underrepresented in sequencing libraries created using the whole genome capture method, they are not absent. Therefore sequencing those samples prepared by whole genome capture to a higher genome wide coverage than is necessary for samples prepared by leukocyte depletion will overcome this bias and allow genotyping of the vast majority of the genome. We therefore recommend for whole genome deep sequencing of *P. vivax*, enough data, taking into account residual contaminating human DNA, should be obtained to achieve 30X-35X coverage of the parasite genome for samples prepared by leukocyte filtration and 50X coverage for samples prepared using the whole genome capture method. For low-pass SNP discovery sequencing, 10X-15X coverage will allow genotyping of a minimum of 65% of the parasite genome using either the standard library preparation method or the whole genome capture protocol (Figure 3).

### SNVs identified in whole genome captured samples

To further evaluate the quality of data that was produced from the whole genome capture method, we next called SNVs from the five whole genome capture samples and compared them to the SNVs previously identified in IQ07 (Table 3). SNVs identified here will be validated and explored in more depth in a future follow-on paper and the validated SNVs will of course be a proportion of those called here using a purely algorithmic approach. We show SNV data here to demonstrate that the whole genome capture method does not produce data with a substantially different number of high confidence algorithmically identified SNVs than the IQ07 suggesting that the sequencing data obtained is of high quality.

**Table 3:** SNVs identifiedby whole genome sequencing

Isolate	Isolate	MSP7 gene family		All MSP gene families		SERA gene family		Metabolic genes	
		**Syn**	**Non-syn**	**Syn**	**Non-syn**	**Syn**	**Non-syn**	**Syn**	**Non-syn**
IQ07^a^	16861	1.82	1.64	2.7	4.1	7.07	5.54	0.45	0.6
Acre3^b^	0	na	na	na	na	na	na	na	na
SA-94	17871	1.93	1.57	3.2	4.41	6.94	5.6	0.37	0.59
SA-95	18916	2.03	1.77	2.48	4.1	7.3	5.03	0.35	0.64
SA-96	17946	1.79	1.81	3.23	4.21	6.64	5.61	0.54	0.7
SA-97	18321	1.86	1.34	2.81	4.19	7.23	5.91	0.44	0.68
SA-98	16596	1.55	1.28	2.89	3.98	6.94	5.04	0.49	0.68

Abbreviations: SNV, single nucleotide variant; Syn, synonymous; Non-syn, non-synonymous; na, not available.

^a^positive control - leukocyte filtered, ^b^negative control.

The total number of SNVs identified in each of the whole genome capture samples (range 16,596 – 18,916) was in concordance with the 16,861 SNVs identified in IQ07. The whole genome capture method performed either on frozen whole blood or mock blood spots did not produce an abnormally high or low number of SNVs indicating that the data was of high quality in line with the data produced by the leukocyte filtration protocol. In total 32,374 SNVs were identified across all five genome capture samples including 98% of the SNVs identified in IQ07.

Next, we analyzed where in the *P.vivax* genome the high confidence SNVs were located. *P. vivax* contains a number of highly variable gene families including the *merozoite surface protein* (*msp*) family and the *serine-rich antigen* (*sera*) family. These gene families are known from *P. falciparum* to be highly variable and initial *P. vivax* resequencing studies have demonstrated a high mutation rate in *P. vivax* as well [4]. In agreement with our previous studies, we find that those gene families predicted to be highly variable do in fact contain high numbers of SNVs per gene in our whole genome captured samples (Table 3). In contrast, annotated metabolic genes have a very low SNV per gene rate, as would be expected of these housekeeping genes.

In addition to being more or less variable, the magnitude of the rate of variability of the gene families analyzed was consistent across all five genome capture samples and the IQ07 positive control. The rate of both synonymous and non-synonymous mutations per gene in the *msp, sera*, and metabolic gene families was found to be a function of the inherent genes in the family and was not affected by the method of sequencing. This result further suggests that the whole genome capture method is unbiased and produces high quality sequencing data that can be used for downstream analysis.

## Discussion

Overall the whole genome capture method provides sufficient data with minimal bias to assign a genotype to greater than 90% of the *P. vivax* genome. Also, the total number of SNVs as well as the SNV rates of gene families was consistent across all five genome capture samples and was in agreement with previous data from IQ07. Using these metrics the whole genome capture technique is as efficient as the current leukocyte filtration method in removing contaminating human genomic DNA and allowing whole genome analysis of the*P. vivax* field samples without introducing any bias into the data.

The critical contribution of this method is to be able to carry out comprehensive whole genome sequencing on *P. vivax* samples where human leukocyte removal has not been or cannot feasibly be done (archival samples or remote sites). In addition, the whole genome capture protocol can be performed on starting bulk gDNA quantities as low as 1 ug. Such analysis will provide new approaches to investigate *P. vivax* population biology with direct applicability to vaccine and drug development.

The cost of sequencing a leukocyte depleted sample and a whole genome captured sample are approximately the same as both methods lead to a mean *P. vivax* DNA percentage of 40%, but the whole genome capture technique increases the cost of library preparation by 100 USD as compared to the leukocyte filtration process. Whole genome capture, though, drastically reduces the time, cost, and effort in the field needed to collect samples because leukocyte filtration requires getting materials and experienced scientists into the field to actually perform the procedure thereby limiting the collection of samples to only certain geographic areas.

Both preparation methods are far superior relative to sequencing extraneous human DNA from a non-leukocyte filtered, non-captured sample. In order to sequence *P. vivax* directly from patient whole blood, 80–100 GB of raw sequencing data would be needed to achieve the desired depth for confident analysis. While sequencing costs continue to come down, at this time it is inefficient to sequence *P. vivax* without using either whole genome capture or leukocyte filtration.

Here we have also estimated, for the first time, the depth of *P. vivax* sequencing that is necessary for resequencing projects. Our data suggest that analyses aimed predominantly at *P. vivax* strain genotyping should collect 30-35X genome-wide coverage, after taking into account contaminating human DNA, for samples prepared by leukocyte filtration and 50X genome-wide coverage for samples prepared by whole genome capture. The marginal benefit of deeper sequencing weighted against cost and throughput efficiency does not support additional sequencing beyond these coverage depths for resequencing studies.

The whole genome capture method is much more amenable to the collection of parasite samples in the field since it can be performed on frozen blood samples or blood spots without the need for complicated laboratory manipulations at the site of collection. Whole genome capture is therefore ideal for analyzing samples collected in resource poor areas such as remote health clinics. Analyzing samples from these areas will greatly expand the knowledge of *P. vivax* population genetics at the local level as well as allow for the tracking of parasite populations.

In addition, whole genome capture is the *only* way to analyze archival samples that were not previously leukocyte filtered before being frozen. Many samples exist around the world that have unique phenotypes but up to this point have only been analyzed using sparse microsatellite markers. The technique presented here allows for more thorough analysis of these samples that to this point would have been cost prohibitive.

## Conclusion

Taken together, the amount of high quality data (over 90% of the genome covered by 5 or more bases) and the expanded range of the whole genome capture technique make this new technique the preferred method for resequencing *P. vivax* field isolates. In addition, studies are on going to expand this technique to whole blood stored on filter paper, which will completely remove the need for a field laboratory thus reducing the technical challenges of collecting *P. vivax* field samples even further.

## Methods

### Ethics statement

The protocol used to collect human blood samples for this work was approved by the Human Subjects Protection Program of The Scripps Research Institute and the University of California, San Diego, and by the Ethical Committees of Universidad Peruana Cayetano Heredia and the Asociacion Benefica PRISMA, Iquitos, Peru. Written informed consent was obtained from each subject or a parent in the case of minors. The consent form states in English and Spanish that samples may be used for any scientific purpose involving this or any other project, now or in the future, and that the samples may be shared with other researchers.

### Sample collection

*P. vivax* DNA used in whole genome capture protocol was isolated from symptomatic Peruvian malaria patients blood smear positive for *P. vivax* malaria. Five to ten mL of whole blood was obtained from each patient with informed consent before anti-malarials were administered. Whole blood samples were centrifuged and the serum and Buffy coat were removed. Erythrocytes were stored at −80 C. IQ07 was collected as described previously and Acre3 was collected in the identical manner [4]. Samples were collected in and around Iquitos, Peru.

### Isolation of genomic DNA

For sample SA96, SA97 and SA98, genomic DNA was isolated from frozen whole blood samples using the DNeasy Blood and Tissue kit (Qiagen) as per the manufacturers instructions. To test the feasibility of capture from DNA isolated from filter paper, 125 ul of SA94 and SA95 were added to Whatman FTA Nucleic Acid storage cards. Cards were left at room temperature for 2–3 weeks before nucleic acid extraction using the Gentra Puregene blood kit (Qiagen).

### Quantification of *P. vivax* DNA with qPCR

A Taqman qPCR assay was designed for both *P. vivax* b-tubulin (PVX_094635) and *P. vivax* ATP-dependent acyl-CoA synthetase (PVX_002785). Primers and probes for each assay are listed in Table S1. The qPCR reaction was conducted using Applied Biosystems Taqman 2x Genotyping Master Mix (Life Technologies), 20 ng bulk genomic DNA, 900 nM of each primer, and 250 nm of the fluorescent hydrolysis probe. Reactions were carried out on an Applied Biosystems StepOne Plus (Life Technologies) using the manufacturers standard protocol. Total *P vivax* DNA was calculated by comparing the Ct value of the sample to a 12-point standard curve of Sal1 reference DNA.

### Creation of whole genome baits (WGB)

Whole genome amplified (Repli-g Kit, Qiagen) Sal1 reference genomic DNA was sheared to an average size of 200 bp using an S-series Covaris Adaptive Focused Acoustic machine (Covaris). Samples underwent end-repair and dA-tailing (New England Biolabs) followed by ligation of Illumina TruSeq v. 3-style Y-adaptors carrying the T7 promoter sequence (Table S1). The T7 ligated library was run on a 2% agarose gel and a band corresponding to 200 bp was cut and purified in 20 ul of TE buffer with MinElute spin columns (Qiagen). Five ul of purified library was then enriched with 14 cycles of PCR using Phusion MasterMix HF (New England Biolabs) (Table S1). Five pmoles of enriched library was used in a 20 ul *in vitro* transcription reaction following the manufacturers protocol (Ambion MEGAshortscript T7 Kit, Life Technologies) with the exception that biotin labeled dUTP was used in replacement of the supplied dUTP. The reaction was purified with an RNeasy Mini column (Qiagen) that included an on column DNase digestion. A single *in vitro* transcription reaction provided a total of 45 ug of Sal1 capture RNA.

### Whole genome capture technique

Bulk genomic DNA was carried through the standard Illumina library preparation process using Adaptive Focused Acoustics for shearing (Covaris), end-repair, A-tailing and ligation (New England Biolabs). Hybridization capture was carried out as previously described [6] with two modifications: 2.5 ug of Human genomic DNA was added in the initial blocking step and only 12 cycles of post capture enrichment were performed. Briefly, 750 ng of the whole genome baits were mixed with 20 units of RNase inhibitor (SUPERase-In, Life Technologies), heated for 2 min at 65 °C in GeneAmp PCR System 9700 thermocycler (Applied Biosystems, Inc), and then mixed with pre-warmed (65 °C) 2× hybridization buffer (Agilent Technologies, Inc.). In a PCR plate, 500 ng of each genomic DNA-fragment library was mixed with 2.5 μg of human Cot-1 DNA, 2.5 μg of salmon sperm DNA, 2.5 ug of Human genomic DNA, and 1 unit of blocking oligonucleotides complementary to the Illumina TruSeq v. 3 adaptor, heated for 5 minutes at 95 °C, and held for 5 minutes at 65 °C in the thermocycler. The mixture was then added to the capture probes, and the solution hybridization was performed for 24 hours at 65 °C.

After the hybridization, the captured targets were selected by pulling down the biotinylated probe/target hybrids by using streptavidin-coated magnetic beads (Dynabeads MyOne Streptavidin T1, Life Technologies). The magnetic beads were prepared by washing 3 times and resuspending in binding buffer (1 M NaCl, 1 mM EDTA, and 10 mM Tris–HCl, pH 7.5). The captured target solution was then added to the beads and rotated for 30 minutes at room temperature. The beads/captured targets were then pulled down by using a magnetic separator, removing the supernatant, resuspending in prewarmed (65 °C) wash buffer (Agilent Technologies, Inc), and then incubated for 15 minutes at room temperature. The beads/captured probes were then pulled down with the magnetic separator and washed by resuspension and incubation for 10 minutes at 65 °C in wash buffer. After three washes, elution buffer (0.1 M NaOH) was added and incubated for 10 minutes at room temperature. The eluted captured targets were then transferred to a tube containing neutralization buffer (1 M Tris–HCl, pH 7.5) and desalted with Agencourt AMPure XP paramagnetic beads (Beckman Coulter). Finally, the targets were enriched by 12-cycle PCR amplification by using 1 μl per sample as a template, and the amplified targets were purified with Agencourt AMPure XP beads.

### Sequencing and data analysis

IQ07 was sequenced as described previously and Acre3 was sequenced in the identical manner [4]. For all of the whole genome capture samples, genomic DNA libraries were sequenced on an Illumina Hi-Seq2000 at the TSRI Next Generation Sequencing Core Facility. Samples were pair end sequenced for 101 bp per read and one 7 bp index read using Illumina v. 3 chemistry. Base calls were made using Illumina RTA (v. 1.12) software. Data for each sample sequenced in this study is available in the NCBI Sequence Read Archive [SRA: SRA047163.1].

Fastq files obtained from sequencing were aligned to the Sal1 reference using BWA (v. 0.5.9) with soft clipping of bases with quality score 2 and below [9]. Data from the same sample library preparation sequenced in different lanes was next merged into one file. PCR duplicates were next identified and marked using Picard (v. 1.51) MarkDuplicates. Aligned reads were then realigned around indels and areas of high entropy using GATK (v. 1.0) IndelRealigner, and the base quality scores of realigned reads were then recalibrated using GATK TableRecalibration [10]. After realignment and recalibration the samples were considered “clean” and ready for use in downstream analysis.

Genome wide coverage and loci covered to a certain percentage were calculated using GATK DepthOfCoverage [10]. For all GATK DepthOfCoverage analyses the minimum mapping quality (mmq) was set to 29 and the minimum base quality (mbq) was set to 20. For downsampling analysis, only reads mapped in proper pairs were considered. These read pairs were filtered out from the total data set using samtools view with the –f 2 option (v. 0.1.16) [12]. Downsampled data sets were created from the properly mapped data sets using Picard DownsampleSam. For each coverage threshold, five randomly downsampled data sets were created and coverage was assessed using GATK DepthOfCoverage. The average of the five technical replicates was used to compare the percent of loci covered by five or more reads between the leukocyte filtered sample and whole genome capture sample.

SNVs were identified using GATK UnifiedGenotyper with the options mbq 20, mmq 29, stand_emit_conf 10, and stand_call_conf 50. Identified SNVs that contained greater than 10% of reads mapping to the reference allele were excluded as false positives. From a total of 125,789 raw SNVs identified across the five whole genome captures samples, 32,372 were retained in the high confidence SNV data set that was used for further analysis.

## Competing interests

The authors declare that they have no competing interests.

## Authors’ contributions

ATB conceived and designed the new method, performed the whole genome capture, prepared samples for sequencing, analyzed the sequencing data and wrote the manuscript. RT designed the new method and created the whole genome baits. SA identified, collected and prepared field samples. RC identified and collected field samples. ALC provided field samples. MUF collected and prepared field samples. NJS provided assistance on designing the whole genome capture method and provided sequencing capacity for field samples. JMV provided field samples and wrote the manuscript. EAW conceived the new method and wrote the manuscript. All authors read and approved the final manuscript.

## Supplementary Material

Additional file 1 Figure S1 and Figure S2. Normalized coverage by GC% content. and comparison of depth of coverage of different %GC regions on chromosome 10. Click here for file
