# Supplementary material for: Whole genome sequencing analysis of Plasmodium vivax using whole genome capture
Source: BMC Genomics. 2012 Jun 21;13:262. doi: 10.1186/1471-2164-13-262 (PMC3410760; doi:10.1186/1471-2164-13-262)
Supplement: Additional file 1 — Figure S1 and Figure S2. Normalized coverage by GC% content. and comparison of depth of coverage of different %GC regions on chromosome 10. [file 1471-2164-13-262-S1.doc]

**Figure S1. Normalized coverage by GC% content.** Normalized coverage ratios for windows of various %GC content. The leukocyte depleted sample (IQ07) and the whole genome capture sample (SA94) perform approximately the same at %GC less than 50%. At higher %GC levels SA94 has much less coverage demonstrating a bias against higher %GC regions of the genome. These regions are still present in the sequencing library but are underrepresented, and therefore the sample must be sequenced to a higher degree to properly analyze these areas.

**Figure S2. Comparison of depth of coverage on chromosome 10.** Two representative regions are highlighted from chromosome 10 showing normalized differential depth of coverage between a sample prepared with leukocyte filtration (IQ07) and a sample prepared using whole genome capture (SA94) based on %GC. (A.) Normalized depth of coverage across a 100 kb region of chromosome 10 that has a %GC content equivalent to the mean of the genome (45%). Both methods are able to efficiently sequence this region with the vast majority of the 100 kb region sequenced to a depth of 20 or greater. (B.) Normalized depth of coverage across a 100 kb region of chromosome 10 that has a %GC content higher than the mean of the genome (55%). The whole genome capture sample (SA94) has substantially less coverage in this 100kb region than the leukocyte filtered sample (IQ07) demonstrating the bias against regions of higher %GC when using the whole genome capture method. While the region is underrepresented in the whole genome capture sample, it is still present at a low level in the sequencing library.
